# Towards the Improved Discovery and Design of Functional Peptides: Common Features of Diverse Classes Permit Generalized Prediction of Bioactivity

**DOI:** 10.1371/journal.pone.0045012

**Published:** 2012-10-08

**Authors:** Catherine Mooney, Niall J. Haslam, Gianluca Pollastri, Denis C. Shields

**Affiliations:** 1 Complex and Adaptive Systems Laboratory, University College Dublin, Belfield, Dublin, Ireland; 2 Conway Institute of Biomolecular and Biomedical Science, University College Dublin, Belfield, Dublin, Ireland; 3 School of Medicine and Medical Science, University College Dublin, Belfield, Dublin, Ireland; 4 School of Computer Science and Informatics, University College Dublin, Belfield, Dublin, Ireland; University of Alberta, Canada

## Abstract

The conventional wisdom is that certain classes of bioactive peptides have specific structural features that endow their particular functions. Accordingly, predictions of bioactivity have focused on particular subgroups, such as antimicrobial peptides. We hypothesized that bioactive peptides may share more general features, and assessed this by contrasting the predictive power of existing antimicrobial predictors as well as a novel general predictor, PeptideRanker, across different classes of peptides.

We observed that existing antimicrobial predictors had reasonable predictive power to identify peptides of certain other classes i.e. toxin and venom peptides. We trained two general predictors of peptide bioactivity, one focused on short peptides (4–20 amino acids) and one focused on long peptides (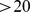 amino acids). These general predictors had performance that was typically as good as, or better than, that of specific predictors. We noted some striking differences in the features of short peptide and long peptide predictions, in particular, high scoring short peptides favour phenylalanine. This is consistent with the hypothesis that short and long peptides have different functional constraints, perhaps reflecting the difficulty for typical short peptides in supporting independent tertiary structure.

We conclude that there are general shared features of bioactive peptides across different functional classes, indicating that computational prediction may accelerate the discovery of novel bioactive peptides and aid in the improved design of existing peptides, across many functional classes. An implementation of the predictive method, PeptideRanker, may be used to identify among a set of peptides those that may be more likely to be bioactive.

## Introduction

Biologically active, or bioactive, peptides encompass a wide range of activities across all kingdoms of life, and the available proteomes of many organisms now represent a rich resource for the computational prediction of potential function of peptides encoded within them. For example, new antibiotic drugs are needed urgently to address the problem of bacterial resistance [1] and bioactive peptides may provide an answer [2], [3]. They may serve as leads for drug design, or in certain circumstances be themselves used as therapeutics. However, bioactive peptides are not only important as a potential source of new antibiotic drugs but have also been shown to have a potential role in the development of new antiviral, antifungal and antiparasitic drugs that may be less susceptible to the development of resistance in pathogens [2]. Bioactive peptides may also modulate human platelet function [4], be used in the development of biomaterials [5] and in wound healing [6]. The identification of food, especially milk, derived bioactive peptides is a growing research area. For example, milk protein derived ACE inhibitors may be added to food with the aim of reducing the risk of developing hypertension [7]. Other bioactive peptides that may be sourced from food include anticancer and antithrombotic peptides [8]. With bioactive peptides showing such potential as new therapeutics, nutraceuticals and functional food ingredients, the discovery and prediction of new bioactive peptides is an increasingly valuable research area.

To date, computational prediction of peptide bioactivity has focused on antimicrobial peptides. The most recent versions of both the antimicrobial peptide database (APD2) [9] and the CAMP database [10] include antiviral, antifungal, antibacterial and antiparasitic peptides. The authors have also studied the amino acid composition of various peptide classes. The experimentally validated CAMP dataset was used to develop prediction tools based on machine learning techniques [10]. Another predictor of antimicrobial peptides, AntiBP2 [11], based on a Support Vector Machine (SVM) was trained on peptides from the APD [12], using the 15 N and C terminal residues and the amino acid composition of the whole peptide. AMPer [13], antimicrobial peptide predictor, used hidden Markov models (HMMs) constructed from known antimicrobial peptides to discover novel antimicrobial peptide candidates (see http://marray.cmdr.ubc.ca/cgi-bin/amp.pl). Another new method for predicting antimicrobial peptides was trained using sequence alignments and feature selection [14].

A number of bioactive peptide databases which cover a range of activities, including, but not limited to antimicrobial peptides, are also available such as BIOPEP [8] and PeptideDB [15]. Although there is some overlap between these databases, they are each focused on particular classes of peptides. BIOPEP is a database of biologically active peptide sequences, and also a tool for the evaluation of proteins as the precursors of bioactive peptides. The peptide activity classes found in BIOPEP include antithrombotic peptides, antiamnestics, celiac toxins, neuropeptides, antibacterial peptides, haemolytic, opioid, heparin binding, anticancer, immunomodulating, antioxidative and peptides labelled as inhibitors, regulating and stimulating. The PeptideDB database includes cytokine and growth factors, peptide hormones, antimicrobial peptides, toxin/venom peptides and antifreeze proteins. However, there are no established prediction methods covering these classes of peptides.

We set out to determine whether it is possible to make useful general predictions regarding peptide bioactivity, or whether predictions are best carried out within particular discrete sub-classes. To assess this, we developed a general bioactive peptide predictor, PeptideRanker, trained in five-fold cross-validation using a novel N-to-1 Neural Network (N1-NN) [16]. This method can prove useful to identify among a set of peptides those that are more likely to be bioactive, allowing the focusing of experimental screening on this subset. Our training set drew from four bioactive peptide databases (BIOPEP, PeptideDB, APD2 and CAMP), covering a diverse set of bioactive peptides. We then investigated how well PeptideRanker can predict different classes of bioactive peptides, and the impact of extracellular status and amino acid composition on predictions.

## Results

### Training a general predictor of peptide bioactivity

In training PeptideRanker to predict bioactive peptides we reduced over-fitting by training and testing using five-fold cross-validation with redundancy reduced datasets and by assessing the performance on two independent datasets. Since evolutionary similarity among peptides in training/test and independent datasets can contribute to over-fitting, we also investigated whether the predictions in the independent dataset relied on sequence similarities. Since our initial investigation indicated that short and long peptide predictions were very different, we developed two separate predictors, one for peptides of twenty residues or less, and one for longer peptides.

**Figure 1:**
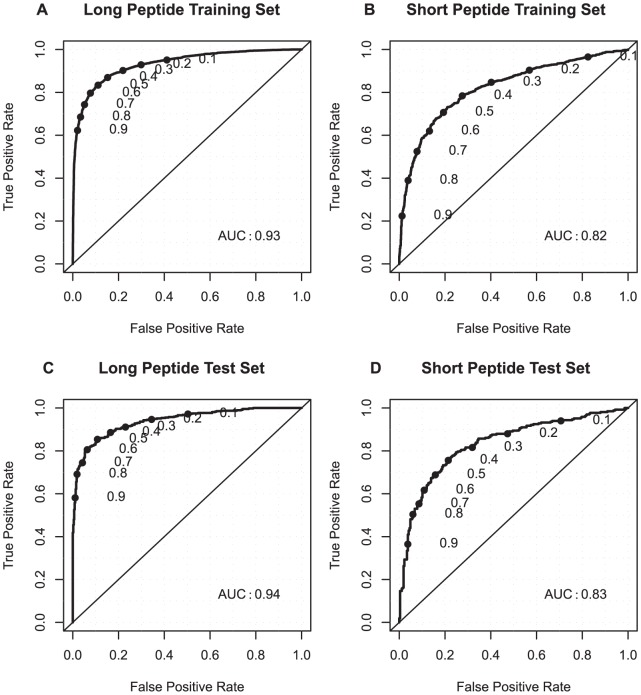
ROC plots for the training and independent test datasets predicted by PeptideRanker. (A) long peptide training dataset (B) short peptide training dataset (C) long peptide independent test dataset (D) short peptide independent test dataset. P-value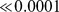 in all four cases.

For every peptide, PeptideRanker predicts the probability (between 0 and 1) of that peptide being bioactive. The closer the predicted probability is to 1, the more confident PeptideRanker is that the peptide is bioactive. The results of five-fold cross-validation for the training dataset, are shown in Table 1 and in Figure 1 as a Receiver Operating Characteristic (ROC) curve with thresholds increasing from 0 to 1, i.e. the cut-off above which a residue is considered to be predicted as a bioactive peptide. We also tested PeptideRanker using two independent test set of peptides with 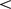
70% sequence similarity to the training set. Table S1 shows the results for PeptideRanker tested on these long and short independent test sets and Figure 1 shows the same results plotted as ROC curves. The results for the independent test sets (where all models of the predictor are ensembled) are slightly better than the results in five-fold cross-validation. The predictor for the long peptides performs better (Matthews Correlation Coefficient (MCC) 0.74 and Area Under Curve (AUC) of 0.94) than the predictor for the short peptides (MCC of 0.54 and AUC of 0.83).

**Table 1:** Performance of PeptideRanker measured in five-fold cross-validation on the training datasets at a threshold of 0.5.

	Long					Short				
	Spec	Sen	FPR	Q	MCC	Spec	Sen	FPR	Q	MCC
Control	84.3	88.9	0.16			73.3	80.6	0.29		
Bioactive	88.3	83.4	0.11			78.4	70.7	0.19		
All				86.1	0.72				75.6	0.51

Specificity (Spec), Sensitivity (Sens) and False Positive Rate (FPR) measured for the bioactive peptide and control peptide classes. Accuracy (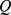) and Matthews Correlation Coefficient (MCC) are measured over all peptides, bioactive peptides and control peptides. See the Materials and Methods section for definitions.

When machine learning methods are used to predict some feature from protein sequences it is common to reduce the sequence similarity within the training set and between the training set and any test sets to 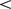
30% sequence similarity. This is essential when, for example, predicting secondary structure from a whole protein sequence where it is known that proteins sharing 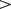
30% sequence similarity are often structurally similar. However, this may not be the case with bioactive peptides, where even one amino acid substitution can change a peptide from bioactive to non-bioactive. Therefore, we considered that redundancy reducing to 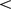
30% sequence similarity would be too strict, and followed previous work where sequences were redundancy reduced to 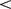
70% sequence similarity. However, we did check for a correlation between prediction accuracy of bioactive peptides in the training and test sets, and the sequence similarity between the test peptides and their most similar peptide in the training set. Linear regression of the prediction versus the sequence similarity indicated the percentage variance (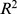) to be only 5% for the long peptides and 1% for the short peptides. This indicates that the predictive power of the method within the independent test dataset is not primarily a result of sequence similarity to peptides in the training set, but mainly arises from other features of the data. We are therefore confident that using training datasets with 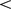
70% sequence similarity between peptides, is appropriate, and is not leading to homology-induced over-fitting of the model.

We estimated the relation between prediction accuracy for the bioactive peptides and peptide length. We found little dependence on length, with 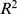 of 0 for the long peptides and 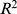 of 0.01 for the short peptides. Thus, within each predictor, the prediction is not strongly influenced by peptide length. During initial training of the predictors (data not shown) the datasets were not split into long and short peptides and we did find in this case that peptide length strongly influenced prediction accuracy which motivated us to split the predictor into long and short peptide predictors. It is clear that the two predictors are behaving in different ways. For example, we found that the predictor of short bioactive peptides is more dependent on amino acid composition than the long peptide predictor, and we discuss this in more detail below.

### Benchmarking against other predictors

We compared the predictive power of PeptideRanker against two state-of-the-art freely available antimicrobial peptide predictors, CAMP [10] and AntiBP2 [11] (Table S1). We used the independent test sets for this comparison which share 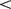
70% sequence similarity with the PeptideRanker training sets but had not been redundancy reduced with respect to the CAMP or AntiBP2 training sets. As AntiBP2 is trained on APD2 peptides [9] and CAMP is trained on peptides from the CAMP database [10], both of which are included in our test set, it is highly likely that at least some of the peptide sequences in this set were used to train CAMP and/or AntiBP2. In effect, this should give CAMP and AntiBP2 an advantage over PeptideRanker when predicting these peptides. However, PeptideRanker is more accurate than either CAMP or AntiBP2 in most cases, which is not surprising as the dataset includes peptides other than antimicrobial peptides, which CAMP and AntiBP2 were not designed to predict.

**Figure 2:**
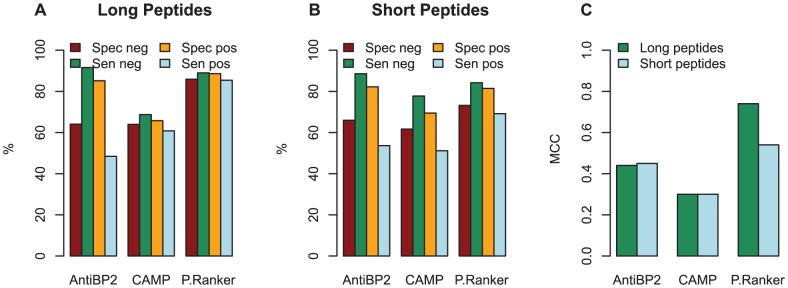
Independent test set. Comparison of PeptideRanker (measured at a threshold of 0.5), CAMP and AntiBP2 tested on the independent test set. (A) long peptides (B) short peptides (C) MCC for long and short peptides. AntiBP2 did not return predictions for 234 (out of a total of 946) of the long and 392 (out of a total of 532) of the short peptides. CAMP did not return predictions for 6 of the long and 5 of the short peptides.

PeptideRanker typically performed better than CAMP (on Specificity (Spec), Sensitivity (Sen), False Positive Rate (FPR), Accuracy (Q) and MCC) for both the long and the short peptides (Figure 2 and Table S1). AntiBP2 had a higher sensitivity on the control peptides and a lower FPR on the bioactive peptides than PeptideRanker for both the long and the short peptides. However, AntiBP2 did not return predictions for 234 of the 946 long peptides and 392 of 532 short peptides. Therefore, AntiBP2 results are not calculated on the whole test set. In fact AntiBP2 only predicted 203 of the 473 true positive long peptides as bioactive, resulting in a low sensitivity on the bioactive peptides and a high FPR for the control set. This imbalance across the bioactive/control peptides is reflected in a low MCC for AntiBP2 of 0.44 compared to the more balanced results for PeptideRanker of 0.74. We see a similar pattern for the short bioactive peptides where only 46 of the 266 bioactive peptides are predicted as bioactive, resulting in a MCC of 0.45 compared to PeptideRanker's MCC of 0.54. Thus, PeptideRanker is better than the antimicrobial predictors at predicting bioactivity across all bioactive peptides. Their relative performances within different classes, including antimicrobial, are investigated further below.

### Importance of amino acid composition

As described in the Datasets section we used two sets of protein sequences from which we randomly selected the control peptides i.e. “Secreted” and “Other”, where “Other” is made up of protein sequences not labelled as secreted or membrane, i.e. non-secreted (see Datasets section for more details). We looked at the amino acid composition of these two sets and the difference between them (Table S2). The most notable difference between the two sets is in the percentage frequency of cysteine which is 2.35% greater in the secreted set, however cysteine is still only the 15th most common residue in the secreted set. The other amino acids with notable differences between the two sets are glutamic acid, lysine, glycine and isoleucine. All other amino acids differ by less than 1%.

**Figure 3:**
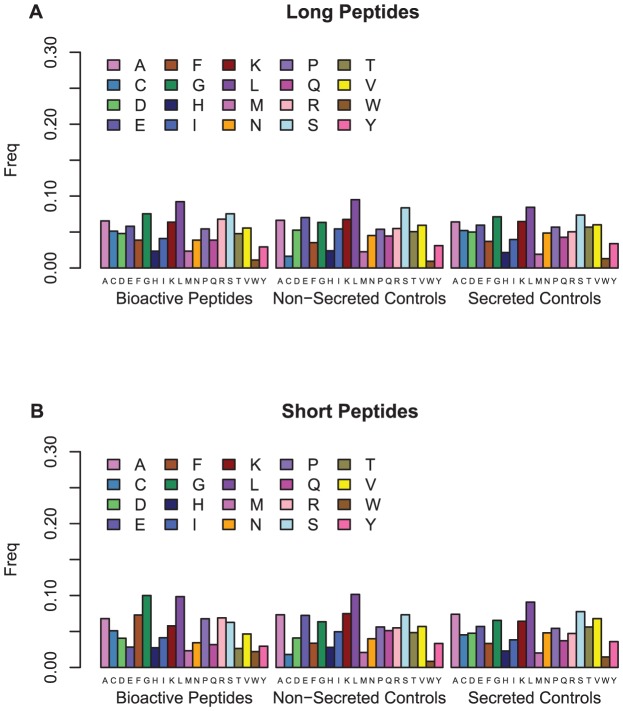
Amino acid content of the bioactive peptides, the non-secreted control peptides and the secreted control peptides in the independent test dataset. (A) long peptides (B) short peptides.

We then looked at the amino acid composition of the long and the short bioactive peptides and the control peptides (secreted and non-secreted) in the independent test sets (Figure 3) and the differences between the bioactive peptides, the secreted and non-secreted control peptides and the secreted and non-secreted sets described above (Table S3). We found very little difference between the short and long randomly selected secreted and non-secreted control peptides. However, there were substantial differences between the long and the short bioactive peptides, with phenylalanine increasing in frequency from 3.9% in the long bioactive peptides to 7.3% in the short bioactive peptides. Similarly, glycine jumps from 7.5% to 10%, whereas glutamic acid and threonine fall from 5.8% and 4.8% to 2.8% and 2.6% respectively. There are also significant differences between the amino acid composition of the bioactive peptides and the secreted and non-secreted control peptides, again especially with the short peptides. Glutamic acid, phenylalanine, glycine and cysteine differ by 4.4%, 3.9%, 3.6% and 3.3% respectively between the short bioactive peptides and the non-secreted control peptides, and phenylalanine, glycine and glutamic acid differ by 4%, 3.5% and 2.9% between the short bioactive peptides and the secreted control peptides. We also compared the differences between the amino acid composition of bioactive peptides and the amino acid composition of the full UniProt secreted protein set. The biggest difference we found for the long bioactive peptides was a decrease from 4.5% to 3.9% for asparagine, and an increase from 5% and 3.9% to 6.8% and 5.1% for arginine and cysteine respectively. We observed greater changes for the short bioactive peptides with threonine and glutamic acid decreasing by 3.3% and 3.1% and phenylalanine and glycine increasing by 3.6% and 2.6% respectively. Overall what is striking is the substantial compositional divergence between the short and long peptide classes.

**Figure 4:**
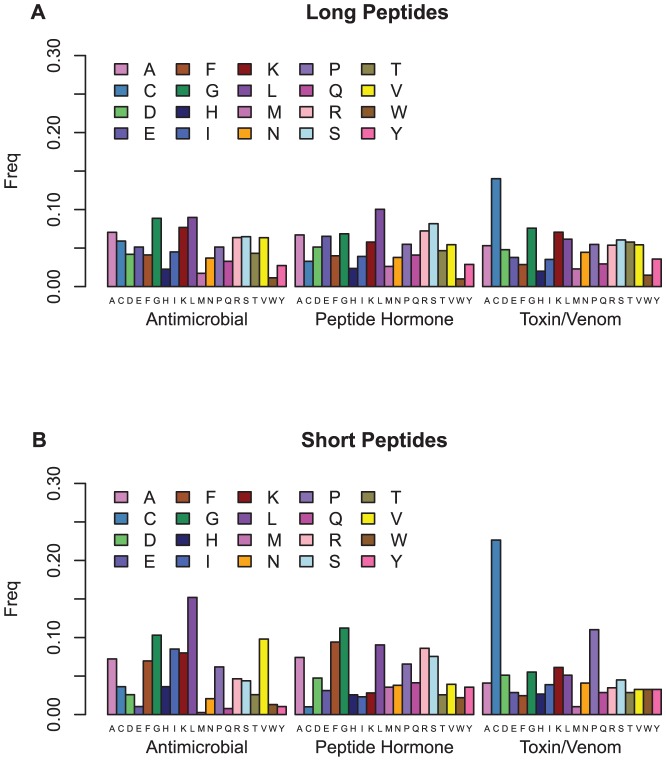
Amino acid content of the three peptide activity classes from the PeptideDB.70 dataset. (A) long peptides (B) short peptides.

Within the two size classes, there is further variation in amino acid frequency that relates to the peptide functional classes. Figure 4 shows the distribution of the amino acid composition across three broad peptide activity classes (antimicrobial, toxin/venom, and peptide hormone). For the long peptides (Figure 4A), cysteine stands out as the most favoured amino acid in the toxin/venom class, whereas it is one of the least favoured in the peptide hormone class. Leucine is the most favoured by the peptide hormones and is also strongly favoured by the antimicrobial peptides along with glycine, which is also favoured by the toxins and venoms. The short peptide classes (Figure 4B) show an even more dramatic shift in their amino acid preferences. Again, leucine is strongly favoured by the antimicrobial peptides but not by the toxins and venoms, which again have a very strong preference for cysteine followed by proline. PeptideRanker may have learned about such clustering of amino acid preferences within particular functional classes, although the training was performed without reference to any such classification of peptide function.

Given this striking difference in amino acid composition, we were interested in investigating to what extent the short and long PeptideRanker predictors make differing use of amino acid composition, in order to identify potentially interesting rules in the discovery and design of bioactive peptides. We inspected the pairwise correlation of the PeptideRanker scores for the long and the short peptides with the frequency of each amino acid (Table S4). For a number of amino acids the correlations showed similar directions e.g. cysteine is markedly favoured by both predictors. Phenylalanine was markedly favoured by the short predictor and disfavoured by the long predictor, with a similar tendency for proline, methionine, glycine and tryptophan; while threonine, valine and glutamic acid were more favoured by the long predictor (Table S4). Again, this justifies the separation into two peptide classes and provides intriguing insights into the differences between amino acid composition of short and long peptides.

Despite the fact that cysteine is not one of the most common amino acids in either the bioactive peptide or the control datasets, the number of cysteines in the peptide has the greatest correlation with the PeptideRanker score (Table S4). To further investigate the role of this important residue we counted the number of sequences with an even number of cysteines in each of our protein sequence datasets. 92% of the UniProt secreted sequences and 90% of “Other” (non-secreted) sequences have at least one cysteine and of these 60% of the secreted sequences have an even number of cysteines compared to 49% of the non-secreted sequences. We then compared these numbers with those for the bioactive peptides in the independent test set. Interestingly, although the average amino acid content of the short and the long peptides is the same (5%) only 20% of short bioactive peptides have at least one cysteine whereas 73% of the long bioactive peptides do. Of these sequences 73% of both sets have an even number of cysteines. We believe that this is due to disulphide bonding including cysteine knot formation [17]. Six cysteines, required to form a cysteine knot, were found in 40% of the long peptides which had an even number of cysteines but in only 23% of the short peptides.

We then broke this down further into the three peptide activity classes. We found that for the toxin and venom peptides 80% of the short and all of the long peptides had at least one cysteine and of these 89% of the short peptides and 81% of the long peptides had an even number of cysteines, and 29% and 53% of these short and long peptides respectively had 6 cysteines. Again, this is likely to be due to cysteine knot formation which is known to be an important motif in toxin peptides [17]. The numbers for the antimicrobial peptides and the peptide hormones are not quite as dramatic with 80%, 60%, 65% and 78% of peptides with cysteines having an even number of cysteines for the long and short antimicrobial peptides and peptide hormones respectively.

To investigate the use of amino acid composition by the predictors, we scrambled the bioactive peptides of the independent test sets, and used them as a new control set. The original bioactive peptide set remained unchanged. We compared the ability of PeptideRanker, AntiBP2 and CAMP to discriminate between the bioactive peptide and the scrambled peptide. It is clear that AntiBP2 and CAMP are mainly relying on amino acid composition, since they do not distinguish well between bioactive peptides and their scrambled sequences (Table S5; MCC values range between −0.01 and 0.06); this is not surprising, given that AntiBP2 specifically relies on amino acid composition for prediction. For PeptideRanker, the MCC is 0.11 for short and 0.34 for long peptides. This indicates that, for PeptideRanker, amino acid composition is an important factor, but by no means the only factor in predicting bioactivity of either short or long peptides.

### Bioactivity prediction is not simply a prediction of extracellular localization

As most bioactive peptides are found in secreted proteins, we selected the control set of peptides for the training dataset from non-secreted protein sequences. This reduces the likelihood of randomly selecting bioactive peptides for the control set by chance. To investigate if the predictors have learned to discriminate only between secreted and non-secreted peptides/proteins, we created a control set of peptides composed of randomly selected sections from a set of proteins known to be secreted. In this situation, by selecting peptides from secreted proteins we increased the chance of erroneously including bioactive peptides in our control peptide sets. However, keeping this in mind, by creating this control set of peptides from proteins known to be secreted, we can still learn something about the tendency to over-predict peptides as bioactive simply because they are from secreted proteins. The results for the three predictors (Table S6). The performance of all three methods is clearly reduced compared to the predictive ability to distinguish bioactive peptides from non-secreted peptides. However, the overall performance of PeptideRanker is substantially better than either of the other two predictors. This indicates that PeptideRanker's ability to predict bioactivity is not solely a matter of predicting a peptide's extracellular features, such as those described above, but also relies on other properties such as the charge distribution of the peptide or the amino acid sequence order.

### Generally predictable features are shared across diverse peptide activity classes

**Figure 5:**
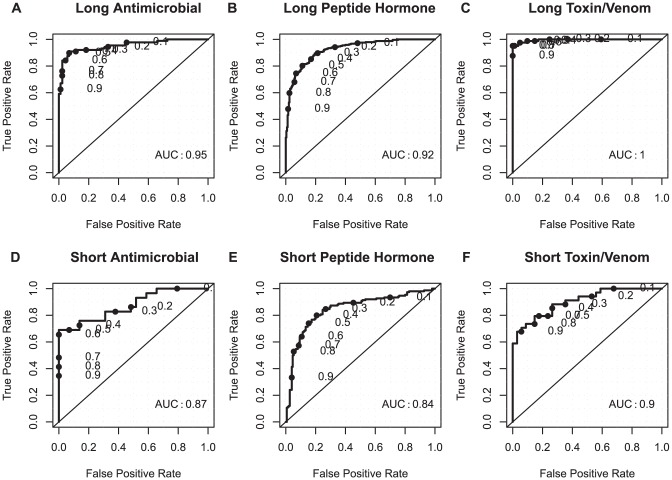
ROC plots for long and short peptide activity classes of the PeptidesDB.70 subset of the independent test set. (A) 88 long antimicrobial peptides (B) 243 long peptide hormones (C) 81 long toxin/venom peptides (D) 29 short antimicrobial peptides (E) 50 short peptide hormones (F) 34 short toxin/venom peptides. P-value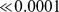 in all six cases.

**Figure 6:**
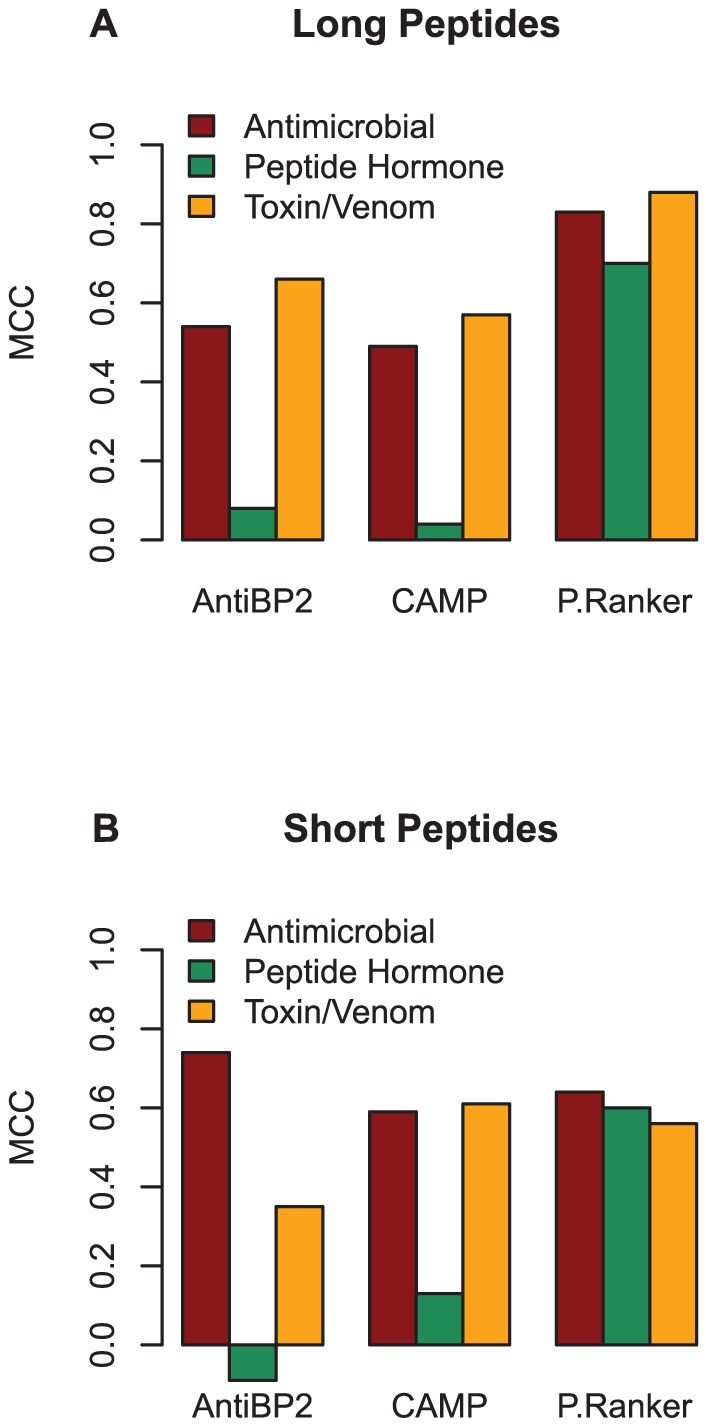
Matthews Correlation Coefficient (MCC) between observed and predicted states for the long and short peptide activity classes. (A) long peptides (B) short peptides.

We investigated whether independent test set peptides of different classes defined by PeptideDB (antimicrobial peptides, peptide hormones and toxin/venom peptides) were differentially predicted by the three methods. ROC curves indicate that all three classes are successfully predicted by PeptideRanker (Figure 5). Performance overall on the antimicrobial peptides for all three predictors is good (Figure 6; Table S7), which is to be expected as AntiBP2 and CAMP are specifically antimicrobial peptide predictors, but neither of them perform well in the peptide hormone class. Both AntiBP2 and CAMP tend to predict the peptide hormones as non-bioactive. In fact, AntiBP2 only predicts 20 of the long and only one of the short peptide hormones as bioactive, corresponding to a FPR (peptides incorrectly predicted as non-bioactive) of 0.85 and 0.96 respectively (Table S8). CAMP performs better, correctly predicting (Sensitivity) 52.9% of the long peptide hormones and 35.3% of the short peptide hormones (FPR of 0.47 and 0.65 respectively). In contrast, PeptideRanker correctly predicts 80.7% of the long peptide hormones and 83% of the short peptide hormones as bioactive. Interestingly, AntiBP2 and CAMP both perform surprisingly well at predicting the toxin and venom peptides, in both the long and short classes (Figure 6; Table S9). In fact, in this case CAMP actually slightly outperforms PeptideRanker. It is important to note that AntiBP2 is unable to predict peptides shorter than 15 amino acids in length, or longer than 100 (see Tables S7, S8 and S9), so that some differences in the performance may be related to differences in the subset investigated.

It can be seen in Table 2 that among short peptides the three groups differ in their charge distributions, with antimicrobial favouring positive charge, while the other two groups include more peptides with overall negative charge. This is also seen for the long peptides. It is of interest to see whether the PeptideRanker scores predicted for short peptides are distinguishing partly on the basis of absolute net charge, since it has been observed that short bioactive peptides tend to favour a high absolute net charge [18]. While among the antimicrobials, the short peptide score is positively correlated with absolute net charge (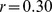), it is negatively correlated for toxin/venom peptides (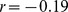) and peptide hormones (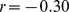). Curiously, the scores of the short peptide hormones are correlated with net charge (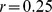). Thus, charge effects may well tend to be class specific.

**Table 2:** Variability in charge across the three peptide activity classes.

	Mean	Mean	Mean absolute	Absolute net
	length	net charge	net charge	charge/length
	Long			
Antimicrobial	60.8	2.9	4.1	0.09
Peptide Hormone	101.7	1.4	4.3	0.05
Toxin/Venom	48.0	1.8	3.4	0.08
	Short			
Antimicrobial	13.6	1.1	1.5	0.12
Peptide Hormone	10.9	0.4	1.2	0.11
Toxin/Venom	14.7	0.3	1.2	0.08

Net charge: sum of positively (K and R) and negatively (D and E) charged residues for each peptide. Absolute net charge: modulus of net charge for each peptide.

Finally, given that there are both common and distinguishing features across the different classes of peptides we wished to examine if it would be possible to predict to which of the three peptide activity classes a bioactive peptide belonged. From the long and short training sets, we extracted all PeptideDB peptide sequences labelled as antimicrobial peptides, peptide hormones and toxin/venom peptides. We trained two predictors, one for long and one for short peptides, using the same architecture as PeptideRanker, except using a three class output where the output predicts the peptide activity as antimicrobial, peptide hormone or toxin/venom. We tested whether or not it was possible for the N-to-1 neural network to learn to discriminate between these three classes (see Table S10). The accuracy of both predictors is very high (85–86%). The least accurately predicted of the three classes is the antimicrobial class, in both cases. It appears that almost all of the false negative antimicrobial peptides are predicted to be peptide hormones (FPR 17–21%).

## Discussion

Our study identifies that bioactivity may be computationally predicted across diverse functional classes of bioactive peptides, and that long peptides (exceeding 20 residues) are best treated as a separate class, compared to short peptides. We observed that features of the sequence of longer peptides contribute to the predictability of peptide bioactivity, whereas in shorter peptides the predictive power is more dominated by amino acid composition. While it is possible that such sequence effects are the result of specific neighbourhood effects within the longer peptides (such as preferred dipeptides or residues located at the two termini) we consider it most likely that this corresponds to the formation of distinct domains within the longer peptides, such as patches of hydrophobicity or charge, representing mini-domains within the longer peptides. The formation of such patches of properties may not be as clearly achievable within shorter peptides.

We propose that the further evaluation of methods to refine the prediction and design of bioactive peptides should consider short (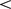
20 residues) and longer peptides as distinct classes, following distinct rules, as we have done here. This has the added advantage that many researchers are interested in either shorter or longer peptides, allowing the development of tools optimized towards these different needs. Our observation that short peptides containing phenylalanine are more likely to be predicted as bioactive, suggests that this amino acid may be particularly useful in conferring bioactivity to shorter peptides; and it is intriguing that longer peptides with phenylalanine are predicted to be bioactive less often. Since longer peptides may adopt internal structure, this difference may indicate that phenylalanine is of most use in forming ligand contacts (typically with larger proteins) than in forming internal structural contacts within the peptide itself. London *et al* [19] noted the strongest enrichment of phenylalanine among peptide interface hotspot residues, consistent with this interpretation. Thus, the two predictors may be distinguishing among different modes of binding, those typical of short peptides and those typical of longer peptides which have more internal structure.

We set out in this study to develop a general predictor of bioactivity, aiming to capture general rules that span across diverse classes of bioactive peptides. In the course of this research, we came to the surprising conclusion that peptide bioactivity predictors trained in one class (antimicrobial) are equally good at predicting toxin/venom peptides. This indicates that these two classes of peptides may share common features, motivating the combination of diverse peptide classes within a single predictor. PeptideRanker provides such a predictor with good performance across different peptide classes. It is likely that the N-to-1 neural network is relying on rules that are general across many peptide classes, augmented by rules that are specific to particular classes. This may explain its superior performance over the two antimicrobial predictors, when predicting bioactivity among peptide hormones. What then is the power of the method to predict peptide bioactivity for a peptide that belongs to a functional class that is not represented within the training set? This can only really be assessed by a formal experimental validation, but we propose that there is sufficient general predictive power encoded within the method to justify its use for the prediction or optimal design of bioactive peptides belonging to functional classes that are not represented in the training set. All predictive methods for peptide bioactivity must be interpreted with caution, since the predictive power is clearly not absolute. We believe that the most useful applications will be in helping to focus experimental decision making, permitting investigators to commence analyses on peptides that are most favoured by the software, leading to overall improved efficiency, in that fewer inactive peptides will be screened. Users need to bear in mind, though, that the method has false negatives as well as false positives, and peptides which are not identified may still be bioactive. Further biophysical characterization of the general sequence and structural features of bioactive peptides may serve to advance the goal of optimizing the prediction and design of peptide bioactivity.

## Materials and Methods

### Datasets

#### Training and independent test dataset

The bioactive peptides used to train PeptideRanker were retrieved from PeptidesDB [15], APD2 [9], CAMP [10] and BIOPEP [8] — in total 18,882 non-unique peptides. The PeptideDB database includes bioactive peptides from animal sources, including cytokine and growth factors, peptide hormones, antimicrobial peptides, toxin/venom peptides, and antifreeze proteins. We included any peptides labelled as peptide hormone, antimicrobial peptide or toxin/venom peptide into the dataset. From BIOPEP we included peptides labelled as antibacterial, antibiotic or anticancer. We included all peptides from CAMP and APD2. A total of 17,532 peptides.

Based on preliminary testing (not shown) we split the dataset into long and short peptides, and trained two independent predictors. We chose a threshold of 20 amino acids based on our subjective idea of what would be considered a short as opposed to a long peptide. 20 amino acids is also the length of the shortest known protein with a stable fold [20]. Splitting the peptides into long (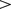
20AA) and short (4–20AA) peptide sets left 13,775 and 3,728 peptides in the long and short sets respectively. We then independently internally redundancy reduced both sets to 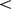
70% sequence similarity using BLAST [21] to create the alignments. As we were searching for short and nearly exact matches we set the word-sizes to 2 (-W 2), low-complexity filter to off (-F F), we set the e-value to 20000 (-e 20000) and used the PAM30 substitution matrix (-M PAM30). This left 4,731 peptides in the long set and 1,330 peptides in the short set.

These two peptide sets were then split into two further sets. One fifth of the short peptides and one tenth of the long peptides were reserved as independent test sets and the remaining peptides became the training set. The training set was then split into five folds i.e. five different sets of peptides where each set is roughly equally sized, disjoint, and their union covers the whole set.

Similar to AntiBP2 [11] the control test set was generated by matching each bioactive peptide with a random peptide from a eukaryotic protein sequence which is known to be intracellular. From the UniProtKB/Swiss-Prot Release 2011_02 we retrieved all eukaryotic Swiss-Prot sequences [22]. We searched these sequences extracting any sequence with a subcellular location annotation, excluding any non-experimental annotations (i.e. those with key words “by similarity”, “probable” or “potential”). We divided these sequences into three sets: “Secreted”, “Membrane” and “Other”. For every peptide in our bioactive set, we randomly selected a sequence from the “Other” set, and then randomly selected a starting position and extracted a peptide of equal length to the bioactive peptide. We then checked if this peptide was found in the list of known bioactive peptides (i.e. the set of 18,882 non-unique peptides). If it was not found we kept this peptide and added it to our control set, otherwise searching again. In this way we have tried to reduce the possibility of including bioactive peptides in our control set, however it is possible that some of our control peptides may be bioactive as they have not been experimentally determined to be non-bioactive. See Table S11 for the number of peptides and average length of peptides in each dataset and Figure S1 for histograms of the length distributions of the four datasets.

In our testing of PeptideRanker we created two further control test sets. In the first case, for all peptides in the independent test set, we generated the corresponding control peptides by scrambling the amino acids of the known bioactive peptides. For the second set, we generated the corresponding control peptides as described above, except that the control peptides were extracted from protein sequences in the “Secreted” set of sequences.

#### Peptide activity classes

From the long and short independent test sets, we extracted all PeptideDB peptide sequences. We refer to this set as the PeptideDB.70 dataset, as no peptide sequence in this dataset has 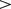
70% sequence similarity to any peptide in the training set. We divided these peptides into three activity subsets i.e. antimicrobial peptides, peptide hormones and toxin/venom peptides (see Table S12). We used the three activity subsets to compare the predictive power of PeptideRanker and two other state-of-the-art antimicrobial peptide predictors in each of these three bioactive peptide classes.

### Predictive architecture: N1-NN

N-to-1 Neural Networks, or N1-NN, have been successfully used to predict the subcellular location of protein sequences [16]. Here, we apply this model to the prediction of peptide bioactivity. The aim of the model is to map a peptide sequence of variable length 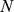 into a single property i.e. bioactive or non-bioactive. Other models tackle this problem at the source, that is, they transform/compress the sequence into a fixed number of descriptors (or into descriptors of pairwise relations between sequences) beforehand, and then map these descriptors into the property of interest. These descriptors are typically frequencies of residues or k-mers, sometimes computed separately on different parts of the sequence [11], [23]. In N1-NN we do not compress the peptide in advance, instead, we decide beforehand only how many features we want to compress a peptide into. These features are stored in a vector 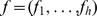, we represent the 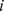-th residue in the peptide as 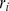, and then 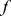 is obtained as:
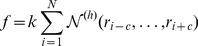
(1)where 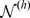 is a non-linear function, which we implement with a two-layered feed-forward Neural Network with 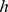 non-linear output units (the peptide-to-feature network). 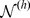 is replicated 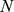 times (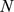 being the peptide length), and 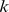 is a normalization constant. The feature vector 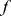 is obtained by combining information coming from all windows of 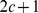 residues in the peptide. In this work the windows have a length of 21 residues for the short peptide predictor i.e. covering the length of the longest peptide, and 41 for the long peptide predictor (see Table S13). The feature vector 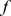 is mapped into the property of interest 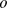 (i.e. bioactive/non-bioactive peptide), as follows:
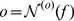
(2)where 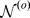 is a non-linear function which we implement with a second two-layered feed-forward neural network (the feature-to-output network). The whole compound neural network (the cascade of 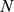 replicas of the peptide-to-feature vector network and one feature-to-output network) is itself a feed-forward neural network and thus can be trained by gradient descent via the back-propagation algorithm. As there are 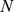 copies of 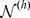 for a peptide of length 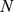, there will be 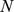 contributions to the gradient for this network, which are simply added together. See [16] for more details on N-to-1 Neural Networks.

#### Training

For each training experiment we implemented two predictors, one for long (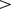
20 amino acids) and one for short bioactive peptides. Each training was conducted in five-fold cross-validation, i.e. five different sets of training runs were performed in which a different fifth of the overall set was reserved for testing and another fifth was reserved for validating. The five fifths were roughly equally sized, disjoint, and their union covered the whole set. The training set was used to learn the free parameters of the network by gradient descent, while the validation set was used to monitor the training process. For each different architecture we ran three trainings (V0, V1, V2), which differed in the number of hidden units in both the peptide-to-feature and the feature-to-output networks, and size of the feature vector (i.e. the number of output units in the peptide-to-feature network) (see Table S13). This ensured that the resulting models were different, which yields larger gains when ensembled.

The weights in the networks were updated every 150 examples (peptides) for the short bioactive peptide predictor and every 500 examples for the long bioactive peptide predictor. 500 epochs of training were performed, which brought the training error to near zero in all cases. Training was performed by gradient descent on the error, which we modelled as the relative entropy between the target class and the output of the network. The overall output of the network (output layer of 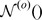) was implemented as a softmax function, while all internal squashing functions were implemented as hyperbolic tangents. The examples were shuffled between epochs. We used a momentum term of 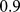 — although this did not significantly affect the final result, it sped up the overall training time by a factor of 10. The learning rate was kept fixed at 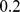 throughout training.

During training the networks that performed best on the validation set were saved, these models were then averaged over the ensemble and evaluated on the corresponding test set. The final results for the five-fold cross-validation are the average of the results on each test set. When testing on an entirely different set from the one used during training (i.e. the independent test set, see the Datasets Section) we ensemble-combined *all* the models from all cross-validation folds of the best architecture.

### Evaluating performance

To evaluate the performance of PeptideRanker we measured Specificity (Spec), Sensitivity (Sens), the True Positive Rate (TPR), the False Positive Rate (FPR), Matthews Correlation Coefficient (MCC) and the Accuracy (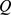) as follows (see [24] for more details):
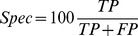


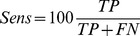


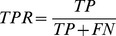


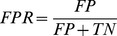


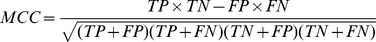


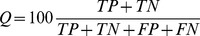
where:

- True Positives (TP): the number of peptides predicted in a class that are observed in that class
- False Positives (FP): the number of peptides predicted in a class that are not observed in that class
- True Negatives (TN): the number of peptides predicted not to be in a class that are not observed in that class
- False Negatives (FN): the number of peptides predicted not to be in a class that are observed in that class

We measure the TPR and FPR as we increase the discrimination threshold from 0 to 1. The results are shown as Receiver Operating Characteristic (ROC) curves where TPR is plotted against FPR. The area under the curve (AUC), which is equivalent to the probability that the classifier will rank a randomly chosen positive instance higher than a randomly chosen negative instance [25], is also shown. The AUC is a number between 0 and 1 inclusive, where 0.5 indicates a random model and 1 is perfect. We used the “verification” package in R [26] to plot the ROC curves and calculate the AUC and to plot the linear regressions from which we derived the correlation, 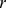, and variance, 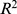 i.e. how well future prediction are likely to be predicted by the model. MCC measures the correlation coefficient between the observed and predicted classifications. A value of 1 represents a perfect prediction, 0 a random prediction and 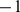 an inverse prediction and is a good indicator of the overall performance of the predictive methods in both bioactive peptide and control peptide classes.

### Implementation

PeptideRanker has been implemented as a web server. The user may submit a list of peptide sequence and PeptideRanker will predict the probability that each of these peptides will be bioactive or not. The list will then be returned to the user ranked by the predicted probability of bioactivity for each peptide. The web server input and output pages are shown in Figure S2 and S3. When the user is interpreting the results is important to note that the server predicts how likely the peptide is to be bioactive, and not how bioactive the peptide is likely to be.

We have chosen as an example the Bovine milk protein beta-casein (UniProt accession number P02666). Beta-casomorphins, which originate from beta-casein, are a group of bioactive peptides [27]. Beta-casomorphin 7 (YPFPGPI) is possibly the most important of these and may have a potential role in human disease such as ischaemic heart disease, diabetes mellitus, sudden infant death syndrome, autism and schizophrenia [27]. This peptide was not included in our training or test sets. We illustrate use of the predictor by examining a series of 7-mer peptides spanning the beta-casomorphin 7 region (Figure S2) and submitted these to PeptideRanker (Figure S3). To run this example on the server click on “example” and then click the “submit” button. Only one of the overlapping peptides scored slightly higher, and it overlaps for 6 of its 7 residues. Many of the overlapping peptides are predicted not to be bioactive, since they are returned with values of under 0.5. (Figure S3). Human beta-casomorphin 7 (YPFVEPI) differs in two positions to the Bovine peptide and is not predicted as strongly to be bioactive (0.506). The other Bovine beta-casomorphins (4, 5, 6, 8 and 11) are also predicted to be bioactive (0.828–0.969). Note that the web server automatically uses the short peptide predictor for peptides of less than 20 amino acids, and the long peptide predictor for peptides of 20 or more residues.

PeptideRanker was trained at a threshold of 0.5 i.e. any peptide predicted over a 0.5 threshold is labelled as bioactive. However, the user may decide to chose a higher threshold to reduce the number of false positives. From our testing (Figure 1) we would expect that choosing a threshold of 0.8 will reduce the false positive rate from 11% and 16% at a 0.5 threshold to 2% and 6% at a 0.8 threshold for long and short peptides respectively. However, increasing the threshold to 0.8 from 0.5 also reduces the true positive rate so the user needs to chose a threshold carefully based on their needs i.e. is it more important to reduce the number of false positives or capture all the true positives?

Another factor that will reduce the number of true positives predicted by PeptideRanker is if the peptide has a cysteine content lower than that of most secreted proteins/peptides i.e. 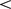
4%. When we tested PeptideRanker we observed that the false negatives in our independent test set (bioactive peptides incorrectly predicted as non-bioactive) had an average cysteine content of just 1.7%.

PeptideRanker was trained and tested on amino acid sequences without any modifications. Peptide synthesis using the 20 natural amino acids has the benefit of drawing on the same set of features as natural proteins and are often relatively inexpensive to synthesise. However, peptide modifications such as the acetylation of the N-terminus, the amidation of the C-terminus, the cyclisation of the peptide or the inclusion of one or more D-amino acids may increase the bioactivity and the specificity of the peptide [28]. The inclusion of D-amino acids may also increase protease resistance [29]. We would suggest that after using PeptideRanker to discover bioactive peptides *in Silico* that various peptide modification would be experimented with *in Vitro* to increase the bioactivity.

PeptideRanker is free for academic use and available at http://bioware.ucd.ie/. The datasets used to train PeptideRanker are available on request.

## Supporting Information

Figure S1 **Histograms of peptide length distribution.** (A) long peptide training set (B) short peptide training set (C) long peptide test set (D) short peptide test set.
(EPS)

Figure S2 **Web server sequence input page.**
(PNG)

Figure S3 **Web server results page.**
(PNG)

Table S1 **Independent test set with control peptide set selected from non-secreted proteins.** Comparison of PeptideRanker (measured at a threshold of 0.5), CAMP and AntiBP2 tested on the independent test set. AntiBP2 did not return predictions for 234 (out of a total of 946) of the long and 392 (out of a total of 532) of the short peptides. CAMP did not return predictions for 6 of the long and 5 of the short peptides.
(PDF)

Table S2 **Percentage amino acid composition of UniProt secreted and non-secreted proteins.**
(PDF)

Table S3 **Difference in amino acid composition between datasets.** Percentage differences in amino acid content between the differente datasets. The first seven columns and the last column are absolute values.
(PDF)

Table S4 **Pairwise correlations between amino acid composition and PeptideRanker scores.** Pearson correlation coefficient for long and short peptides between amino acid composition and PeptideRanker scores, and the difference (Diff) between long and short correlations.
(PDF)

Table S5 **Independent test set with a control peptide set of scrambled bioactive peptides.** Comparison of PeptideRanker (measured at a threshold of 0.5), CAMP and AntiBP2 tested on the independent test set. The control peptides are generated by scrambling the bioactive peptide set i.e. the amino acid composition of both the control and the bioactive set is the same. AntiBP2 did not return predictions for 234 of the long and 393 of the short peptides. CAMP did not return predictions for 12 of the long and 8 of the short peptides.
(PDF)

Table S6 **Independent test set with control peptide set from secreted proteins.** Comparison of PeptideRanker (measured at a threshold of 0.5), CAMP and AntiBP2 tested on the independent test set, with control peptides randomly selected from secreted proteins. AntiBP2 did not return predictions for 223 of the long and 394 of the short peptides. CAMP did not return predictions for 11 of the long and 7 of the short peptides.
(PDF)

Table S7 **Prediction of antimicrobial peptides.** Comparison of AntiBP2, CAMP and PeptideRanker tested on the PeptideDB.70 Antimicrobial peptide activity class subset of the independent test set. AntiBP2 did not return predictions for 20 of the 176 long and 34 of the 58 short peptides. CAMP did not return a prediction for one of the long peptides. Statistics were calculated on the subset of peptides for which predictions were available.
(PDF)

Table S8 **Prediction of peptide hormones.** Comparison of AntiBP2, CAMP and PeptideRanker tested on the PeptideDB.70 Peptide hormone activity class subset of the independent test set. AntiBP2 did not return predictions for 212 of the 486 long and 254 of the 300 short peptides. CAMP did not return predictions for five of the long peptides. Statistics were calculated on the subset of peptides for which predictions were available.
(PDF)

Table S9 **Prediction of toxin/venom peptides.** Comparison of AntiBP2, CAMP and PeptideRanker tested on the PeptideDB.70 Toxin/Venom peptide activity class subset of the independent test set. AntiBP2 did not return predictions for 38 of the 68 short peptides. CAMP did not return a prediction for one of the short peptides. Statistics were calculated on the subset of peptides for which predictions were available.
(PDF)

Table S10
**Performance of three class peptide activity predictor measured in five-fold cross-validation on the peptide activity subsets of the full training datasets at a threshold of 0.5.** The number of peptides per class (Num), Specificity (Spec), Sensitivity (Sens), Matthews Correlation Coefficient (MCC) and False Positive Rate (FPR) measured for the three peptide activity classes. Accuracy (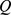) and Generalised Correlation (GC) are measured over all peptides. See the Materials and Methods section for definitions.(PDF)

Table S11 **Number of peptides and average peptide length per class.**
(PDF)

Table S12 **Number of bioactive and control peptides per activity class.** Number of bioactive and control peptides per activity class in the PeptideDB.70 subset of the independent test sets (see Figures 4 and 5).
(PDF)

Table S13
**Network parameters.** 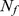: size of the feature vector; 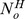: number of hidden units in the feature-to-output network; 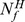: number of hidden units in the peptide-to-feature network; 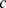 is the context i.e. 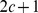 is the size of the window being considered.(PDF)
