# Supplementary figures and images for: Towards the Improved Discovery and Design of Functional Peptides: Common Features of Diverse Classes Permit Generalized Prediction of Bioactivity
Source: PLoS One. 2012 Oct 8;7(10):e45012. doi: 10.1371/journal.pone.0045012 (PMC3466233; doi:10.1371/journal.pone.0045012)

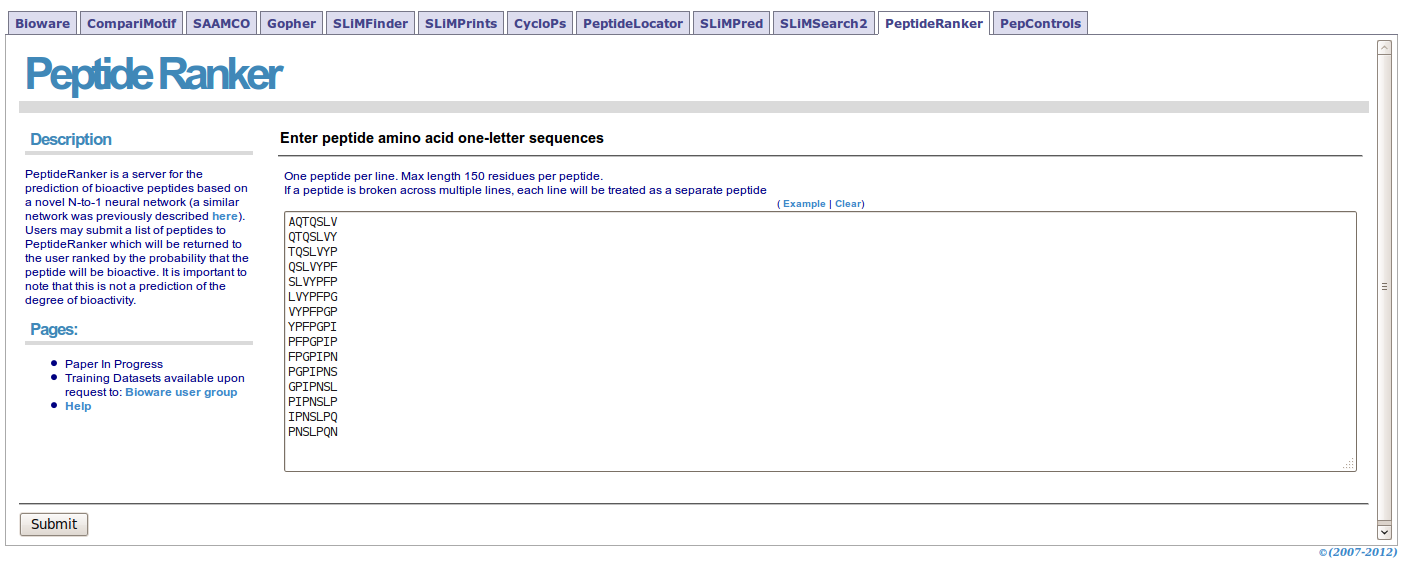

Supplement: Figure S2 — Web server sequence input page. (PNG) [file pone.0045012.s002.png]

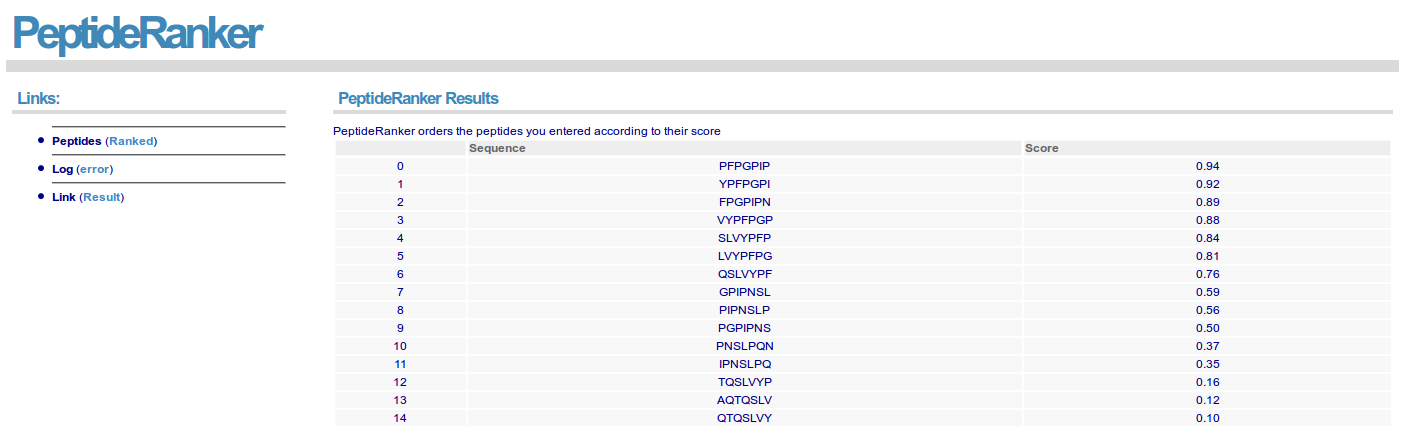

Supplement: Figure S3 — Web server results page. (PNG) [file pone.0045012.s003.png]
