# Supplementary material for: Towards the Improved Discovery and Design of Functional Peptides: Common Features of Diverse Classes Permit Generalized Prediction of Bioactivity
Source: PLoS One. 2012 Oct 8;7(10):e45012. doi: 10.1371/journal.pone.0045012 (PMC3466233; doi:10.1371/journal.pone.0045012)
Supplement: Table S1 — Independent test set with control peptide set selected from non-secreted proteins. Comparison of PeptideRanker (measured at a threshold of 0.5), CAMP and AntiBP2 tested on the independent test set. AntiBP2 did not return predictions for 234 (out of a total of 946) of the long and 392 (out of a total of 532) of the short peptides. CAMP did not return predictions for 6 of the long and 5 of the short peptides. (PDF) [file pone.0045012.s004.pdf]

Table S1. Independent test set with control peptide set selected from non-secreted proteins

	Long					Short				
	Spec	Sen	FPR	Q	MCC	Spec	Sen	FPR	Q	MCC
					AntiBP2					
Non-secreted control	64.1	**91.8**	0.51			66.0	**88.6**	0.47		
Bioactive	85.2	48.5	**0.08**			**82.2**	53.6	**0.11**		
All				70.0	0.44				71.1	0.45
					CAMP					
Non-secreted control	64.0	68.7	0.39			61.7	77.8	0.49		
Bioactive	65.7	60.8	0.31			69.4	51.2	0.22		
All				64.8	0.30				64.4	0.30
					PeptideRanker					
Non-secreted control	**85.9**	89.0	**0.15**			**73.2**	84.2	**0.31**		
Bioactive	**88.6**	**85.4**	0.11			81.4	**69.2**	0.16		
All				**87.2**	**0.74**				**76.7**	**0.54**

Comparison of PeptideRanker (measured at a threshold of 0.5), CAMP and AntiBP2 tested on the independent test set. AntiBP2 did not return predictions for 234 (out of a total of 946) of the long and 392 (out of a total of 532) of the short peptides. CAMP did not return predictions for 6 of the long and 5 of the short peptides.
