# Supplementary material for: Towards the Improved Discovery and Design of Functional Peptides: Common Features of Diverse Classes Permit Generalized Prediction of Bioactivity
Source: PLoS One. 2012 Oct 8;7(10):e45012. doi: 10.1371/journal.pone.0045012 (PMC3466233; doi:10.1371/journal.pone.0045012)
Supplement: Table S2 — Percentage amino acid composition of UniProt secreted and non-secreted proteins. (PDF) [file pone.0045012.s005.pdf]

Table S2. Percentage amino acid composition of UniProt secreted and non-secreted proteins

	Secreted	Non-secreted	Diff
A	6.75	6.75	0
C	3.93	1.58	2.35
D	5.20	5.20	0
E	5.91	7.14	-1.23
F	3.74	3.74	0
G	7.43	6.15	1.28
H	2.34	2.45	-0.11
I	4.28	5.37	-1.09
K	5.61	6.80	-1.19
L	8.78	9.25	-0.47
M	2.05	2.23	-0.18
N	4.47	4.49	-0.02
P	5.81	5.35	0.46
Q	4.23	4.56	-0.33
R	5.00	5.67	-0.67
S	7.67	8.19	-0.52
T	5.92	5.26	0.66
V	6.13	5.88	0.25
W	1.37	1.00	0.37
Y	3.35	2.92	0.43
