# Supplementary material for: Towards the Improved Discovery and Design of Functional Peptides: Common Features of Diverse Classes Permit Generalized Prediction of Bioactivity
Source: PLoS One. 2012 Oct 8;7(10):e45012. doi: 10.1371/journal.pone.0045012 (PMC3466233; doi:10.1371/journal.pone.0045012)
Supplement: Table S3 — Difference in amino acid composition between datasets. Percentage differences in amino acid content between the differente datasets. The first seven columns and the last column are absolute values. (PDF) [file pone.0045012.s006.pdf]

Table S3. Difference in amino acid composition between datasets

	Bioactive long-Bioactive short	Non-secreted long-Non-secreted short	Secreted long-Secreted short	Bioactive long-Non-secreted long	Bioactive long-Secreted long	Bioactive short-Non-secreted short	Bioactive short-Secreted short	Avg Secreted Freq-Bioactive long	Avg Secreted Freq-Bioactive short	Uniprot Secreted-Uniprot non-Secreted
A	0.2	0.7	1	0.1	0.1	0.5	0.6	-0.2	0	0
C	0	0.2	0.7	3.5	0.1	3.3	0.6	1.2	1.2	2.4
D	0.7	1.2	0.3	0.5	0.2	0	0.7	-0.4	-1.1	0
E	3	0.2	0.3	1.2	0.2	4.4	2.9	-0.1	-3.1	1.2
F	3.4	0.2	0.4	0.3	0.2	3.9	4	0.1	3.6	0
G	2.5	0	0.6	1.2	0.4	3.6	3.5	0.1	2.6	1.3
H	0.4	0.4	0.1	0	0.2	0.1	0.4	0	0.4	0.1
I	0	0.5	0.1	1.4	0.1	0.8	0.3	-0.2	-0.2	1.1
K	0.6	0.7	0.1	0.4	0.1	1.7	0.6	0.8	0.2	1.2
L	0.6	0.6	0.6	0.3	0.8	0.3	0.8	0.4	1.1	0.5
M	0	0.2	0.1	0.1	0.4	0.3	0.3	0.3	0.3	0.2
N	0.5	0.5	0	0.6	1	0.6	1.4	-0.6	-1	0
P	1.3	0.2	0.2	0.1	0.3	1.1	1.3	-0.4	1	0.5
Q	0.7	0.7	0.6	0.6	0.4	1.9	0.5	-0.4	-1.1	0.3
R	0.1	0	0.3	1.3	1.8	1.4	2.2	1.8	1.9	0.7
S	1.3	1	0.4	0.8	0.2	1.1	1.5	-0.1	-1.4	0.5
T	2.1	0.2	0	0.3	0.9	2.2	3	-1.1	-3.3	0.7
V	0.9	0.3	0.8	0.4	0.4	1	2.1	-0.6	-1.5	0.2
W	1.1	0.1	0.1	0.2	0.2	1.4	0.8	-0.3	0.8	0.4
Y	0	0.2	0.2	0.2	0.5	0.4	0.6	-0.4	-0.4	0.4

Percentage differences in amino acid content between the differente datasets. The first seven columns and the last column are absolute values.
