# Supplementary material for: Towards the Improved Discovery and Design of Functional Peptides: Common Features of Diverse Classes Permit Generalized Prediction of Bioactivity
Source: PLoS One. 2012 Oct 8;7(10):e45012. doi: 10.1371/journal.pone.0045012 (PMC3466233; doi:10.1371/journal.pone.0045012)
Supplement: Table S4 — Pairwise correlations between amino acid composition and PeptideRanker scores. Pearson correlation coefficient for long and short peptides between amino acid composition and PeptideRanker scores, and the difference (Diff) between long and short correlations. (PDF) [file pone.0045012.s007.pdf]

**Table S4. Pairwise correlations between amino acid composition and PeptideRanker scores**

| | Long | Short | Diff |
|---|---|---|---|
| T | 0.0300 | -0.1752 | -0.2052 |
| V | -0.0159 | -0.1948 | -0.1789 |
| E | -0.1212 | -0.2913 | -0.1701 |
| K | 0.1093 | 0.0297 | -0.0796 |
| S | -0.0439 | -0.1112 | -0.0673 |
| R | 0.0013 | -0.0627 | -0.0640 |
| C | 0.4676 | 0.4043 | -0.0633 |
| H | -0.1788 | -0.2111 | -0.0323 |
| D | -0.1612 | -0.1900 | -0.0288 |
| Q | -0.0746 | -0.1012 | -0.0266 |
| L | -0.0428 | -0.0587 | -0.0159 |
| N | -0.0217 | -0.0367 | -0.0150 |
| I | -0.0272 | -0.0339 | -0.0067 |
| Y | 0.0046 | 0.0082 | 0.0036 |
| A | -0.0927 | -0.0578 | 0.0349 |
| W | 0.0454 | 0.1382 | 0.0928 |
| G | 0.0272 | 0.1412 | 0.1140 |
| M | -0.0834 | 0.0370 | 0.1204 |
| P | -0.0196 | 0.1270 | 0.1466 |
| F | -0.1530 | 0.1699 | 0.3229 |

Pearson correlation coefficient for long and short peptides between amino acid composition and PeptideRanker scores, and the difference (Diff) between long and short correlations.
