# Supplementary material for: Towards the Improved Discovery and Design of Functional Peptides: Common Features of Diverse Classes Permit Generalized Prediction of Bioactivity
Source: PLoS One. 2012 Oct 8;7(10):e45012. doi: 10.1371/journal.pone.0045012 (PMC3466233; doi:10.1371/journal.pone.0045012)
Supplement: Table S5 — Independent test set with a control peptide set of scrambled bioactive peptides. Comparison of PeptideRanker (measured at a threshold of 0.5), CAMP and AntiBP2 tested on the independent test set. The control peptides are generated by scrambling the bioactive peptide set i.e. the amino acid composition of both the control and the bioactive set is the same. AntiBP2 did not return predictions for 234 of the long and 393 of the short peptides. CAMP did not return predictions for 12 of the long and 8 of the short peptides. (PDF) [file pone.0045012.s008.pdf]

Table S5. Independent test set with a control peptide set of scrambled bioactive peptides

	Long					Short				
	Spec	Sen	FPR	Q	MCC	Spec	Sen	FPR	Q	MCC
AntiBP2										
Scrambled control	49.9	50.8	0.51			50.0	45.7	0.47		
Bioactive	49.7	48.7	0.49			48.7	52.9	0.54		
All				49.8	0.00				49.3	-0.01
CAMP										
Scrambled control	50.4	39.8	0.39			53.1	55.4	0.49		
Bioactive	50.3	60.8	0.60			53.4	51.2	0.45		
All				50.3	0.01				53.2	0.06
PeptideRanker										
Scrambled control	75.7	45.5	0.15			57.3	41.4	0.31		
Bioactive	61.0	85.4	0.55			54.1	69.2	0.59		
All				65.4	0.34				55.3	0.11

Comparison of PeptideRanker (measured at a threshold of 0.5), CAMP and AntiBP2 tested on the independent test set. The control peptides are generated by scrambling the bioactive peptide set i.e. the amino acid composition of both the control and the bioactive set is the same. AntiBP2 did not return predictions for 234 of the long and 393 of the short peptides. CAMP did not return predictions for 12 of the long and 8 of the short peptides.
