# Supplementary material for: Towards the Improved Discovery and Design of Functional Peptides: Common Features of Diverse Classes Permit Generalized Prediction of Bioactivity
Source: PLoS One. 2012 Oct 8;7(10):e45012. doi: 10.1371/journal.pone.0045012 (PMC3466233; doi:10.1371/journal.pone.0045012)
Supplement: Table S6 — Independent test set with control peptide set from secreted proteins. Comparison of PeptideRanker (measured at a threshold of 0.5), CAMP and AntiBP2 tested on the independent test set, with control peptides randomly selected from secreted proteins. AntiBP2 did not return predictions for 223 of the long and 394 of the short peptides. CAMP did not return predictions for 11 of the long and 7 of the short peptides. (PDF) [file pone.0045012.s009.pdf]

Table S6. Independent test set with control peptide set from secreted proteins

	Long					Short				
	Spec	Sen	FPR	Q	MCC	Spec	Sen	FPR	Q	MCC
					AntiBP2					
Secreted control	59.6	73.0	0.51			60.5	71.0	0.47		
Bioactive	63.6	48.7	0.27			64.3	52.9	0.29		
All				60.9	0.22				62.0	0.24
					CAMP					
Secreted control	61.3	62.0	0.39			59.2	70.7	0.49		
Bioactive	61.5	60.8	0.38			63.5	51.2	0.29		
All				61.4	0.23				60.9	0.22
					PeptideRanker					
Secreted control	77.9	51.4	0.15			70.2	72.5	0.31		
Bioactive	63.7	85.4	0.49			71.6	69.2	0.27		
All				68.4	0.39				70.9	0.42

Comparison of PeptideRanker (measured at a threshold of 0.5), CAMP and AntiBP2 tested on the independent test set, with control peptides randomly selected from secreted proteins. AntiBP2 did not return predictions for 223 of the long and 394 of the short peptides. CAMP did not return predictions for 11 of the long and 7 of the short peptides.
