# Supplementary material for: Towards the Improved Discovery and Design of Functional Peptides: Common Features of Diverse Classes Permit Generalized Prediction of Bioactivity
Source: PLoS One. 2012 Oct 8;7(10):e45012. doi: 10.1371/journal.pone.0045012 (PMC3466233; doi:10.1371/journal.pone.0045012)
Supplement: Table S7 — Prediction of antimicrobial peptides. Comparison of AntiBP2, CAMP and PeptideRanker tested on the PeptideDB.70 Antimicrobial peptide activity class subset of the independent test set. AntiBP2 did not return predictions for 20 of the 176 long and 34 of the 58 short peptides. CAMP did not return a prediction for one of the long peptides. Statistics were calculated on the subset of peptides for which predictions were available. (PDF) [file pone.0045012.s010.pdf]

Table S7. Prediction of antimicrobial peptides

	Long					Short				
	Spec	Sen	FPR	Q	MCC	Spec	Sen	FPR	Q	MCC
					AntiBP2					
Control	69.2	92.3	0.42			90.9	83.3	0.09		
Bioactive	88.2	58.4	0.08			83.3	90.9	0.17		
All				75.4	0.54				87.1	0.74
					CAMP					
Control	72.6	78.4	0.30			84.0	72.4	0.14		
Bioactive	76.3	70.1	0.22			75.8	86.2	0.28		
All				74.3	0.49				79.3	0.59
					PeptideRanker					
Control	90.1	93.2	0.10			75.0	93.1	0.31		
Bioactive	92.9	89.8	0.06			90.9	69.0	0.06		
All				91.5	0.83				81.0	0.64

Comparison of AntiBP2, CAMP and PeptideRanker tested on the PeptideDB.70 Antimicrobial peptide activity class subset of the independent test set. AntiBP2 did not return predictions for 20 of the 176 long and 34 of the 58 short peptides. CAMP did not return a prediction for one of the long peptides. Statistics were calculated on the subset of peptides for which predictions were available.
