# Supplementary material for: Towards the Improved Discovery and Design of Functional Peptides: Common Features of Diverse Classes Permit Generalized Prediction of Bioactivity
Source: PLoS One. 2012 Oct 8;7(10):e45012. doi: 10.1371/journal.pone.0045012 (PMC3466233; doi:10.1371/journal.pone.0045012)
Supplement: Table S8 — Prediction of peptide hormones. Comparison of AntiBP2, CAMP and PeptideRanker tested on the PeptideDB.70 Peptide hormone activity class subset of the independent test set. AntiBP2 did not return predictions for 212 of the 486 long and 254 of the 300 short peptides. CAMP did not return predictions for five of the long peptides. Statistics were calculated on the subset of peptides for which predictions were available. (PDF) [file pone.0045012.s011.pdf]

Table S8. Prediction of peptide hormones

	Long					Short				
	Spec	Sen	FPR	Q	MCC	Spec	Sen	FPR	Q	MCC
					AntiBP2					
Control	51.5	90.5	0.85			48.8	91.3	0.96		
Bioactive	60.6	14.6	0.09			33.3	4.4	0.09		
All				52.6	0.08				47.8	-0.09
					CAMP					
Control	52.7	51.4	0.47			54.3	76.7	0.65		
Bioactive	51.6	52.9	0.49			60.2	35.3	0.23		
All				52.2	0.04				56.0	0.13
					PeptideRanker					
Control	82.1	88.9	0.19			77.0	84.7	0.25		
Bioactive	87.9	80.6	0.11			83.0	74.7	0.15		
All				84.8	0.70				79.7	0.60

Comparison of AntiBP2, CAMP and PeptideRanker tested on the PeptideDB.70 Peptide hormone activity class subset of the independent test set. AntiBP2 did not return predictions for 212 of the 486 long and 254 of the 300 short peptides. CAMP did not return predictions for five of the long peptides. Statistics were calculated on the subset of peptides for which predictions were available.
