# Supplementary material for: Towards the Improved Discovery and Design of Functional Peptides: Common Features of Diverse Classes Permit Generalized Prediction of Bioactivity
Source: PLoS One. 2012 Oct 8;7(10):e45012. doi: 10.1371/journal.pone.0045012 (PMC3466233; doi:10.1371/journal.pone.0045012)
Supplement: Table S9 — Prediction of toxin/venom peptides. Comparison of AntiBP2, CAMP and PeptideRanker tested on the PeptideDB.70 Toxin/Venom peptide activity class subset of the independent test set. AntiBP2 did not return predictions for 38 of the 68 short peptides. CAMP did not return a prediction for one of the short peptides. Statistics were calculated on the subset of peptides for which predictions were available. (PDF) [file pone.0045012.s012.pdf]

Table S9. Prediction of toxin/venom peptides

	Long					Short				
	Spec	Sen	FPR	Q	MCC	Spec	Sen	FPR	Q	MCC
					AntiBP2					
Control	76.5	92.6	0.28			63.2	80.0	0.47		
Bioactive	90.6	71.6	0.07			72.7	53.3	0.20		
All				82.1	0.66				66.7	0.35
					CAMP					
Control	70.8	92.6	0.38			77.8	84.9	0.24		
Bioactive	89.3	61.7	0.07			83.9	76.5	0.15		
All				77.2	0.57				80.7	0.61
					PeptideRanker					
Control	98.6	88.9	0.01			78.8	76.5	0.20		
Bioactive	89.9	98.8	0.11			77.1	79.4	0.23		
All				93.8	0.88				77.9	0.56

Comparison of AntiBP2, CAMP and PeptideRanker tested on the PeptideDB.70 Toxin/Venom peptide activity class subset of the independent test set. AntiBP2 did not return predictions for 38 of the 68 short peptides. CAMP did not return a prediction for one of the short peptides. Statistics were calculated on the subset of peptides for which predictions were available.
