# Supplementary material for: Towards the Improved Discovery and Design of Functional Peptides: Common Features of Diverse Classes Permit Generalized Prediction of Bioactivity
Source: PLoS One. 2012 Oct 8;7(10):e45012. doi: 10.1371/journal.pone.0045012 (PMC3466233; doi:10.1371/journal.pone.0045012)
Supplement: Table S10 — Performance of three class peptide activity predictor measured in five-fold cross-validation on the peptide activity subsets of the full training datasets at a threshold of 0.5. The number of peptides per class (Num), Specificity (Spec), Sensitivity (Sens), Matthews Correlation Coefficient (MCC) and False Positive Rate (FPR) measured for the three peptide activity classes. Accuracy () and Generalised Correlation (GC) are measured over all peptides. See the Materials and Methods section for definitions. (PDF) [file pone.0045012.s013.pdf]

Table S10. Performance of three class peptide activity predictor measured in five-fold cross-validation on the peptide activity subsets of the full training datasets at a threshold of 0.5

	Long					Short				
	Num	Spec	Sen	MCC	FPR	Num	Spec	Sen	MCC	FPR
Antimicrobial	797	79.1	68.9	0.67	5	916	65.4	53.7	0.53	5.0
Peptide Hormone	2186	88.6	93.3	0.77	17.3	2790	90.1	93.7	0.72	24.1
Toxin/Venom	728	86.9	85	0.83	3.2	866	79.2	76.0	0.73	3.7
All	3711	Q	86.4	GC	0.76	4572	Q	85.4	GC	0.66

The number of peptides per class (Num), Specificity (Spec), Sensitivity (Sens), Matthews Correlation Coefficient (MCC) and False Positive Rate (FPR) measured for the three peptide activity classes. Accuracy (Q) and Generalised Correlation (GC) are measured over all peptides. See the Materials and Methods section for definitions.
