# Supplementary material for: Towards the Improved Discovery and Design of Functional Peptides: Common Features of Diverse Classes Permit Generalized Prediction of Bioactivity
Source: PLoS One. 2012 Oct 8;7(10):e45012. doi: 10.1371/journal.pone.0045012 (PMC3466233; doi:10.1371/journal.pone.0045012)
Supplement: Table S11 — Number of peptides and average peptide length per class. (PDF) [file pone.0045012.s014.pdf]

Table S11. Number of peptides and average peptide length per class

	Training/Test Set		Independent Test Set	
	Long	Short	Long	Short
Control	4258	1064	473	266
Bioactive	4258	1064	473	266
Avg. length	78.0	12.1	75.6	12.0
