# Supplementary material for: Towards the Improved Discovery and Design of Functional Peptides: Common Features of Diverse Classes Permit Generalized Prediction of Bioactivity
Source: PLoS One. 2012 Oct 8;7(10):e45012. doi: 10.1371/journal.pone.0045012 (PMC3466233; doi:10.1371/journal.pone.0045012)
Supplement: Table S12 — Number of bioactive and control peptides per activity class. Number of bioactive and control peptides per activity class in the PeptideDB.70 subset of the independent test sets (see Figures 4 and 5). (PDF) [file pone.0045012.s015.pdf]

Table S12. Number of bioactive and control peptides per activity class

	Long			Short		
	Bioactive	Control	Total	Bioactive	Control	Total
Antimicrobial	88	88	176	29	29	58
Peptide Hormone	243	243	486	150	150	300
Toxin/Venom	81	81	162	34	34	68
Total	412	412	824	213	213	426

Number of bioactive and control peptides per activity class in the PeptideDB.70 subset of the independent test sets (see Figures 4 and 5).
