# Supplementary material for: Towards the Improved Discovery and Design of Functional Peptides: Common Features of Diverse Classes Permit Generalized Prediction of Bioactivity
Source: PLoS One. 2012 Oct 8;7(10):e45012. doi: 10.1371/journal.pone.0045012 (PMC3466233; doi:10.1371/journal.pone.0045012)
Supplement: Table S13 — Network parameters. : size of the feature vector; : number of hidden units in the feature-to-output network; : number of hidden units in the peptide-to-feature network; is the context i.e. is the size of the window being considered. (PDF) [file pone.0045012.s016.pdf]

**Table S13. Network parameters**

| | Long | | | Short | | |
|---|---|---|---|---|---|---|
| | V0 | V1 | V2 | V0 | V1 | V2 |
| $N_f$ | 9 | 10 | 11 | 5 | 6 | 7 |
| $N_o^H$ | 9 | 10 | 11 | 5 | 6 | 7 |
| $N_f^H$ | 9 | 10 | 11 | 5 | 6 | 7 |
| $c$ | 20 | 20 | 20 | 10 | 10 | 10 |

$N_f$: size of the feature vector; $N_o^H$: number of hidden units in the feature-to-output network; $N_f^H$: number of hidden units in the peptide-to-feature network; $c$ is the context i.e. $2c+1$ is the size of the window being considered.
